# Chimeric Antigen Receptor T Cells Targeting NKG2D-Ligands Show Robust Efficacy Against Acute Myeloid Leukemia and T-Cell Acute Lymphoblastic Leukemia

**DOI:** 10.3389/fimmu.2020.580328

**Published:** 2020-12-15

**Authors:** Lina Driouk, Joanina K. Gicobi, Yusuke Kamihara, Kayleigh Rutherford, Glenn Dranoff, Jerome Ritz, Susanne H. C. Baumeister

**Affiliations:** ^1^ Division of Hematologic Malignancies, Dana-Farber Cancer Institute, Boston, MA, United States; ^2^ Department of Biostatistics, Harvard T.H. Chan School of Public Health, Boston, MA, United States; ^3^ Novartis Institutes of Biomedical Research, Cambridge, MA, United States; ^4^ Harvard Medical School, Boston, MA, United States; ^5^ Division of Pediatric Oncology, Dana-Farber Cancer Institute, Boston, MA, United States; ^6^ Division of Pediatric Hematology-Oncology, Boston Children’s Hospital, Boston, MA, United States

**Keywords:** CAR T cells, acute myeloid leukemia, T cell acute lymphoblastic leukemia, NKG2D-ligands, NKG2D activating receptor

## Abstract

CAR T cell approaches to effectively target AML and T-ALL without off-tumor effects on healthy myeloid or T cell compartments respectively are an unmet medical need. NKG2D-ligands are a promising target given their absence on healthy cells and surface expression in a wide range of malignancies. NKG2D-ligand expression has been reported in a substantial group of patients with AML along with evidence for prognostic significance. However, reports regarding the prevalence and density of NKG2D-ligand expression in AML vary and detailed studies to define whether low level expression is sufficient to trigger NKG2D-ligand directed CART cell responses are lacking. NKG2D ligand expression in T-ALL has not previously been interrogated. Here we report that NKG2D-ligands are expressed in T-ALL cell lines and primary T-ALL. We confirm that NKG2D-ligands are frequently surface expressed in primary AML, albeit at relatively low levels. Utilizing CAR T cells incorporating the natural immune receptor NKG2D as the antigen binding domain, we demonstrate striking *in vitro* activity of CAR T cells targeting NKG2D-ligands against AML and T-ALL cell lines and show that even low-level ligand expression in primary AML targets results in robust NKG2D-CAR activity. We found that NKG2D-ligand expression can be selectively enhanced in low-expressing AML cell lines and primary AML blasts *via* pharmacologic HDAC inhibition. Such pharmacologic NKG2D-ligand induction results in enhanced NKG2D-CAR anti-leukemic activity without affecting healthy PBMC, thereby providing rationale for the combination of HDAC-inhibitors with NKG2D-CAR T cell therapy as a potential strategy to achieve clinical NKG2D-CAR T cell efficacy in AML.

## Introduction

Acute myeloid leukemia (AML) is the most common adult acute leukemia and carries a poor prognosis (1, 2). Pediatric AML has a better prognosis but relapse occurs in about 30% of patients (3). Similarly, T-cell acute lymphoblastic leukemia (T-ALL) is characterized by a high relapse rate and poor overall survival which dramatically declines with patient age (4). Advances in chimeric antigen receptor (CAR) T cell-therapy have led to remarkable clinical remissions in refractory B-cell lineage leukemias and lymphomas (5–10). However, similar approaches to target AML and T-ALL have been challenged by the expression of target antigens on normal myeloid precursors and normal T cells. In AML, candidate antigens such as CD123 (11), folate-receptor β (12) or FLT-3 are expressed early in myeloid differentiation and CAR T-cell mediated targeting may result in significant and potentially permanent myeloablation (13). While rescue and consolidation *via* an allogeneic stem cell transplant is possible, this is associated with added risk of morbidity and mortality. Similarly, candidate antigens such as CD33 (14, 15) are expressed on healthy myeloid progenitors and raise concern about hepatotoxicity given expression on hepatic Kupffer cells and the occurrence of veno-occlusive disease following treatment with CD33-directed toxin-conjugated antibodies (16). Targeting of T-ALL with lineage-restricted antigens is inherently complicated by the potential for T-cell fratricide. Innovative approaches to prevent CART-fratricide, by eliminating target antigen expression on the effector CAR T cells have been reported (17, 18). However, these are not protective of native T cells and T-cell aplasia carries a greater infectious risk than CD19-associated B-cell aplasia, which is manageable with administration of therapeutic immunoglobulins.

Rather than targeting a single lineage-associated antigen, we explored targeting a group of inducible ligands of the activating immune receptor NKG2D, namely, MICA, MICB and the UL16-binding proteins (ULBP) 1–6. NKG2D-ligands are upregulated in response to DNA damage, inflammation and malignant transformation (19). NKG2D-ligand expression has been reported in a number of solid tumors and hematologic malignancies, while ligands are generally absent on healthy tissues (20–22). In previous studies we focused on a novel CAR which uses the naturally occurring NKG2D receptor as the antigen-binding domain fused to the intracellular domain of CD3ζ. In contrast to native NKG2D which provides only a TCR-dependent costimulatory signal in CD8 T cells and is predominantly expressed among CD8 T cells, expression of the NKG2D-CAR mediates direct T-cell activation upon recognition of NKG2D-ligands independent of a TCR-based signal in both CD4 and CD8 T cells. In murine models, NKG2D-CAR T cells demonstrated efficacy in eradicating established multiple myeloma (MM), lymphoma and ovarian cancers and inducing autologous immunity protective against tumor re-challenge after NKG2D-CAR T cells were no longer detectable (23–29). Subsequently, other groups demonstrated preclinical efficacy in models of osteosarcoma (30), triple negative breast-cancer (31) and gastric cancer (32). Furthermore, NKG2D-CAR T cells were effective against tumors with heterogeneous ligand expression (33) and NKG2D-CAR-expressing NK cells eradicated myeloid suppressor cells in the tumor microenvironment of solid tumors (34). Importantly, human NKG2D-CAR T cells do not react to autologous peripheral blood mononuclear cells (PBMCs) or bone marrow (BM) from healthy donors *in vitro* (24). Nevertheless, reports of low level NKG2D-ligand expression in gut epithelium (35, 36), the possibility of NKG2D-ligand-upregulation in healthy tissues under conditions of cell stress and infection (19) and dose-dependent toxicity observed in mouse models (37, 38) were of potential concern for the translation of this approach into the clinic (39). In the first-in human Phase 1 study of NKG2D-CAR T cells in patients with AML and multiple myeloma, no safety or feasibility concerns were raised, but a clinical efficacy signal was not seen (40). The 7 AML patients enrolled on the study all expressed at least one NKG2D-ligand in the AML blast population, however the mean fluorescence intensity (MFI) of expression was low and no comprehensive studies to assess the preclinical efficacy of NKG2D-CAR T cells in AML or T-ALL have been conducted.

While the role of NKG2D-ligands in T-ALL has not been characterized, NKG2D-ligand expression has been reported in a substantial group of patients with AML (22, 41–43). Furthermore, there is evidence for clinical significance of NKG2D-ligand expression in AML with impact on survival and relapse (44). However, NKG2D-ligands in AML are not consistently and often weakly expressed (45), and detailed studies to define whether low level expression is sufficient to trigger NKG2D-CAR T cell responses were lacking. NKG2D-ligands are regulated *via* the ATM/ATR pathway and may be selectively induced on AML blasts *via* pharmacologic mechanisms such as HDAC-inhibition. In several studies, this led to enhanced susceptibility of AML blasts to NK-cell mediated killing (46, 47). Epigenetic therapeutics such as HDAC-inhibitors, which are used clinically with a well characterized safety profile in a number of clinical trials in AML (48–50), represent attractive candidates for synergistic combinations with immunotherapies. Here we comprehensively interrogate the extent of NKG2D-ligand expression in AML and T-ALL and the associated efficacy of NKG2D-CART cells in functional readouts. We confirm that NKG2D-ligand expression is frequently weak in primary AML but nevertheless leads to a robust functional response mediated by NKG2D-CAR T cells. Furthermore, we demonstrate that selective NKG2D-ligand upregulation *via* pharmacologic HDAC inhibition enhances NKG2D-CAR T efficacy against AML, thereby supporting this strategy for combination therapy.

## Materials and Methods

### Generation of Viral Vectors

Healthy donor PBMC was obtained and RNA stabilized in RNAprotect Cell Reagent (Qiagen). RNA was isolated using RNAeasy Plus Mini Kits (Qiagen). High-capacity cDNA reverse transcription kits (Applied Biosystems) were used to generate cDNA per manufacturer’s instructions. The following primers were used to amplify NKG2D and CD3ζ chain coding sequences (NKG2D: FV: ATGGGGTGGATTCGTGGTCG RV: TTACACAGTCCTTTGCATGC, CD3ζ: FV: ATGAAGTGGAAGGCGCTTTTCACC) using Expand High Fidelity PCR Systems (Roche). PCR products were cloned into the pCR4-TOPO plasmid vector using TOPO-TA cloning kits (Invitrogen, Life technologies), transformed into One shot TOP10 E. coli cells (Invitrogen, Life technologies) using the OneShot chemical transformation protocol per manufacturer’s instructions and plated on Ampicillin-containing LB Plates. Colonies were then PCR-screened and grown in LB Broth containing 100 ng/ml Ampicillin for 16 h at 37°C. Plasmid DNA was isolated using QIAprep Spin Miniprep Kits (Quiagen) and sequences verified using *Sanger* Sequencing. Restriction-site cloning was used to ligate the sequence of the cytoplasmic domain of CD3ζ chain into the γ-retroviral vector pMFG separated by an IRES sequence from green fluorescent protein (GFP). The full-length sequence of the NKG2D-gene (KLRK1) was then fused to the CD3ζ cytoplasmic domain separated by the Xho-I site, followed by the IRES-GFP sequence using In-Fusion Dry-Down PCR Cloning Kits (Clontech), and transformed into Stellar Competent Cells (Clontech), using chemical transformation and plated on Ampicillin-containing LB agar plates. As a control, pMFG vector was generated containing GFP only in similar fashion. Clones were grown in LB Broth containing Ampicillin and the sequence confirmed by *Sanger* sequencing following Maxi-Prep isolation of DNA-plasmid (Quiagen). Plasmid DNA was transfected into the 293GPG packaging cell line using Lipofectamine 2000 (Invitrogen) and Opti-mem media (Invitrogen). 293GPG cells were maintained in DMEM media containing FCS, Pen/Strep, Hepes and l-glutamine but lacking tetracycline to facilitate production of VSV-G pseudotyped viral particles. Viral supernatant was collected daily while 293GPG cells were viable. Viral particles were concentrated by ultracentrifugation (Beckman) and cryopreserved at −80°C. Concentrated virus was used to infect the PG13 γ-retroviral packaging cells (ATCC) in the presence of polybrene. Stable high-titer PG13 cell lines were infected with either pMFG.CD3ζNKG2D-IRES-GFP or pMFG.GFP and grown in DMEM 10%FCS/1%Pen/Strep. Viral supernatants were collected, filtered using 0.45 μm Durapore 50 ml vacuum driven filtration systems (Steriflip, Millipore) and either stored at −80°C or used fresh.

### T Cell Transduction

T cells were generated from healthy donor PBMC (40 × 10^6^) isolated by Ficoll-density separation and stimulated with CD3/CD28 Dynabeads (Invitrogen) at a ratio of 1:1 in 40 ml complete X-VIVO (cX-VIVO, X-VIVO-15 Gentamicin and Phenol Red containing media (Lonza) with 5% hAB serum (Gemini)) in the presence of 100 IU/ml interleukin-2 (IL-2) (Proleukin, Bayer) in T75 flasks for 4 days. T cells at a concentration of 1 × 10^6^/ml with CD3/CD28 Dynabeads were transduced in viral-vector loaded 24-well plates (Corning) on days 4 and 5 and fed on day 6 as described (51), before being harvested on day 7, stained with DAPI and sorted based on DAPI negative/GFP+ T cells on a M Aria II SORP (BD). Sorted cells were then expanded for an additional 3 to 4 days in cX-VIVO containing 5 ng/ml IL-7 and 2.5 ng/ml IL-15 (both Peprotech) at 0.25 × 10e6/ml in in GREX 10 culture flasks (Wilson Wolf) or 24 well tissue-culture treated plates (Corning).

### Cells

Primary human AML and T-ALL bone marrow and peripheral blood specimens were obtained after informed, written consent under IRB-approved protocols, Ficoll-isolated and cryopreserved until use. The following mycoplasma-tested cell lines were cultured in RPMI 10% Fetal Calf Serum and 1% Penicillin and Streptomycin: Molm13, MV-411, HL-60 (AML), Jurkat, HPB-ALL, KOPT-K1, DND-41 (T-ALL), K562 and B16.

### T Cell Functional Assays

#### CD107a Degranulation and Intracellular Cytokine Production

Target- and effector cells were plated in cX-VIVO at 1 × 10^6^/ml in duplicate or triplicate at 100 μl/well in 96-well round-bottom plates (Corning) at a ratio of 1:1 in the presence of anti-CD28 (BD, L293), anti-CD49d (BD, L25) and Golgi-Stop (BD) and Golgi-Plug (BD) at a final concentration of 1 μl/ml and 5 μl of CD107a mAb (BD, H4A3) for 5 h at 37°C. In experiments utilizing blocking mAbs, NKG2D-CAR T cells were incubated with purified anti-human CD314 (20 μg/ml, BD, 1D11) or isotype mAb (20 μg/ml) for 15 min prior to coculture. Cells were surface-stained with anti-NKG2D (Biolegend, 1D11), CD3 (BD, SK7), CD4 (BD, RPA-T4) and CD8 mAbs (BD, RPA-T8), fixed and permeabilized using Cytofix/Cytoperm kits (BD) per manufacturer’s instructions followed by intracellular staining with anti-TNFα (Biolegend, Mab11) and IFNγ (BD, B27) mAbs or respective isotypes before analysis by flow cytometry. Effector cells were incubated with Staphylococcal Endotoxin (Toxin Technology) as a positive control.

#### Cytotoxicity

Target cells were labeled with Calcein AM (Invitrogen) at 0.5 × 10^6^/ml in cX-VIVO for 30 min on ice in the dark, washed, adjusted to 1 × 10^5^/ml, plated at 100 μl/well in duplicate at varying effector:target ratios in a total volume of 200 μl/well and incubated for 6 h at 37°C. For minimum and maximum controls, target cells were incubated without effector cells or with 1% Saponin (Sigma) respectively. Cells were washed and stained with 7AAD (BD) for 15 min on ice, prior to analysis by flow cytometry. Percentage specific lysis was calculated as follows: %SL= [(% cytolysis sample − % cytolysis minimum)/(% cytolysis maximum − % cytolysis minimum)] × 100. Percentage cytolysis was determined by (1 - % live cells). % live cells were defined as % of GFP- Calcein AM+ targets which stained negative for 7AAD.

#### Proliferation

Target cells were irradiated at a dose of 100 Gy in HBSS and subsequently washed and adjusted at 1 × 10^6^ cells/ml in X-VIVO 5%hABS media. T cells were labeled with CellTrace Violet dye (Life Technologies) at 1 × 10^6^ cells/ml in PBS for 20 min at 37°C protected from light before adding 3 ml of X-VIVO 5%hABS media and incubating for 5 min. Cells were pelleted by centrifugation, resuspended in fresh pre-warmed media for 10 min, quenched with 3 ml cold media, spun and adjusted at 1 × 10^6^ cells/ml in X-VIVO 5% hABS media. T cells (5 × 10^4^ cells/well) were then incubated with the respective target cells at a 1:1 ratio, alone or with 25 ng/ml IL-15 (Peprotech) in a total volume of 200 μl/well × 3 days. Cells were then washed, stained, fixed and analyzed by flow cytometry with CountBright beads (Thermofisher) to determine the number of proliferating cells.

#### Co-culture

Effector and target cells were resuspended in cX-VIVO at 1 × 10^6^/ml, plated in duplicate or triplicate at a ratio of 1:1 in a volume of 200 μl/well in 96-well round bottom plates (Corning) and incubated at 37°C × 24 h. Cell-free supernatant was harvested and analyzed using Human IFN-γ OPTEIA ELISA kits (BD) per manufacturer’s instructions on a SpectraMax M3 instrument.

### Flow Cytometry

Monoclonal antibodies (mAbs) were used to define blast populations as follows: AML: CD45 (BD, Hl30), CD34 (BD, 581/CD34), CD117 (Invitrogen,104D2), HLA-DR (BD, G46-6) and CD33 (Biolegend, WM53). T-ALL: CD45, CD5 (Biolegend, L17F12), CD7 (BD, M-T701), CD2 (BD, RPA-2.10), CD3 (BD, SK7) and CD8 (BD, RPA-T8). The following mAbs were used to define T cell subsets: CD3(BD, UCHT1), CD4 (BD, RPA-T4), CD8 (BD, RPA-T8)), CD45RO (BD, UCHL1), CD62L (BD, DREG-56), CD95 (Biolegend, DX2). RhNKG2D Fc chimera (R&D, 12990NK-050) and rhIgG1 Fc (R&D, 110-HG-100) were PE-conjugated per manufacturer instructions (Abcam) and titrated prior to use. For individual NKG2D-ligand detection the following mAbs were used: MICA (MBL, AMO1), MICB (MBL, BMO1) (both conjugated per manufacturer instructions, Abcam), ULBP-1 (170818), ULBP2/5/6 (165903), ULBP3 (166510), ULBP4 (709116) (all R&D). Cells were washed and stained in phosphate-buffered saline supplemented with 2% fetal calf serum (Gibco) at 4°C after blocking with FcR blocking reagent (Miltenyi). LIVE/DEAD^®^ Fixable Aqua Dead Cell Stain (Invitrogen, L34966) was used for live/dead staining. When applicable, cells were fixed using Cytofix kits (BD). Flow cytometry was performed using 4-Laser M Fortessa Analyzers (BD). Flow cytometric analysis was performed using FlowJo V10 (Tree Star).

### Pharmacologic Upregulation

AML cell lines were treated with valproic acid (VPA), azaciditine (Aza), hydroxyurea (HU), or the bromodomain inhibitor JQ1 at a range of different concentrations and durations as indicated at 37°C and 5% CO_2_. Freshly thawed primary AML bone marrow samples were treated with 1 mM VPA for 24 h at 37°C and 5% CO_2_. VPA sodium salt (Sigma Aldrich) was dissolved in sterile PBS immediately before being added to the cell suspensions and an equal volume of PBS was added to the negative control. After thawing, AML samples were washed twice with pre-warmed complete StemPro-34 (for upregulation experiments) or cX-VIVO (for simple ligand staining). Cell clumps were dissolved using 100 μl DNAase and 50 μl 1 M MgCl_2_ solution in 10 ml of the medium.

Cell lines were treated with 1 mM VPA in RPMI-1640 with 10%FCS/1% Pen/Strep in either flat-bottom 24-well tissue culture plates or T75 tissue culture flasks at a concentration of 5 × 10^5^/ml. Primary AML bone marrow samples were cultured for 24 h in round-bottom 96-well tissue culture plates in the presence or absence of 1 mM VPA at a concentration of 1 × 10^6^/ml in complete StemPro-34 serum-free medium (Gibco) with 2 mM l-glutamine supplemented with the following growth factors: SCF and Flt3-ligand at 100 ng/ml each, GM-CSF at 20 ng/ml and IL-3 and IL-6 at 10 ng/ml each (all Peprotech). Cells were washed in media before use in staining and functional studies.

### Mass Cytometry (CyTOF)

Freshly manufactured NKG2D-CAR T cells or Empty-control T cells and K562 target cells were resuspended at 10 × 10^6^/ml in cX-VIVO and T cells either cultured alone or with K562 targets at a 1:1 ratio in a volume of 200 μl in 96-well round-bottom plates (Corning) for 24 h at 37°C in 50 wells per condition. Cells from each condition were subsequently pooled, spun, and cryopreserved in Bambanker solution (Lymphotec Inc.). Aliquots of all tested conditions were thawed, processed and analyzed in parallel. Cells were incubated with ^103^Rh Cell-ID Intercalator (Fluidigm) for 15 min per manufacturer’s instructions, fixed with Cytofix Fixation Buffer, processed and analyzed on a Helios mass cytometer (Fluidigm) as previously described (52). A detailed listing of antibodies is provided in **Table 1**. T-cell phenotypic populations were defined as follows: T_Naive_ (CD45RA+, CCR7+, CD95−), T_SCM_ (CD45RA+, CCR7+, CD95+), T_CM_ (CD45RA-, CCR7+, CD95+), T_EM_ (CD45RA−, CCR7−, CD95+), T_TEMRA_ (CD45RA+, CCR7−).

**Table 1:** Summary of antibodies and metal tags used for mass cytometry.

Summary of monoclonal antibodies and metal tags used for mass cytometry				
TARGET	SPECIES	CLONE	ISOTYPE	MANUFACTURER
CD45	Human	HI30	89Y	Fluidigm
CD3	Human	UCHT1	154Sm	Fluidigm
CD4	Human	SK3	174Yb	Fluidigm
CD8	Human	SK1	168Er	Fluidigm
CD25	Human	2A3	149Sm	Fluidigm
CD45RA	Human	Hl100	169Tm	Fluidigm
CCR7	Human	G043H7	159Tb	Fluidigm
CD95	Human	DX2	164Dy	Fluidigm
NKG2D	Human	1D11	166Er	BD Biosciences
ICOS	Human	C398.4A	148Nd	Fluidigm
CXCR3	Human	G025H7	163Dy	Fluidigm
4-1BB	Human	4B4-1	173Yb	Fluidigm
OX40	Human	ACT35	150Nd	Fluidigm
LAG-3	Human	11C3C65	165Ho	Biolegend
Ki67	Cross	B56	161DY	Fluidigm

Preconjugated antibodies were purchased from Fluidigm. All other antibodies were purchased in carrier-protein-free PBS and conjugated with the respective metal isotype using the MaxPAR antibody conjugation kit (Fluidigm) according to manufacturer’s instructions. Metal-labeled antibodies were diluted to 0.5 mg/ml in Candor PBS Antibody Stabilization solution (Candor Bioscience GmbH) for long-term storage at 4°C.

## Results

### Transgene and NKG2D-CAR T Cell Characteristics

**Figure 1:**
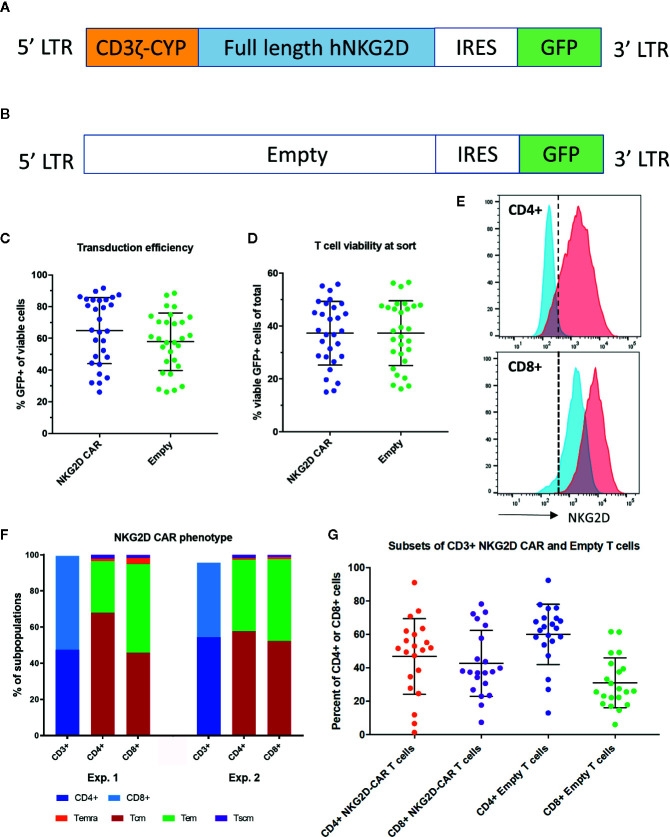
Transgene and NKG2D-CAR T cell characteristics. **(A)** The transgene of the NKG2D-CAR vector includes the cytoplasmic domain of the CD3ξ chain, full-length human NKG2D and is separated from GFP by an IRES domain. **(B)** The transgene of the Empty control vector contains GFP only **(C)** The transduction efficiency expressed as %GFP+ cells among viable cells is shown for NKG2D-CAR and Empty control T cells. Bars represent Mean ± Standard Deviation (SD) **(D)** No differences in viability of transduced T cells were detected between the NKG2D-CAR and Empty control vector cultures at the time of sorting, when evaluating % viable GFP+ cells among total cells in the original T cell culture. Bars represent Mean ±SD **(E)** NKG2D-CAR expression was also measured by NKG2D-staining demonstrating minimal NKG2D-expression in CD4+ T cells transduced with Empty control vector (blue) and only native NKG2D-expression at levels expected in CD8+ T cells. In contrast CD4+ T cells transduced with NKG2D-CAR vector (red) demonstrated NKG2D-surface expression in CD4+ T cells and a shift in MFI beyond native NKG2D-expression in CD8+ T cells **(F)** Two representative examples depicting the phenotypic subset composition of NKG2D-CAR T cells are shown **(G)**. CD4:8 ratio of NKG2D-CAR and Empty control T cells in the CD4+ and CD8+ subsets are shown respectively. Bars represent Mean ± SD.

We generated a NKG2D-CAR T cell construct based on the γ-retroviral vector pMFG, fusing full length NKG2D to the cytoplasmic CD3ξ domain and containing GFP in the transgene (CAR) (**Figure 1A**) as well as a control vector containing GFP only (Empty) (**Figure 1B**). Freshly isolated T cells were transduced either with CAR or Empty with a mean transduction efficiency (% GFP+ of viable cells) of 64.9% (SD 20.9) for NKG2D-CAR T cells and 57.9% (SD 18.2) for Empty control T cells (n=29 each) (**Figure 1C**). There was no significant difference in transduced viable cells (% viable, GFP+ cells of total cells in the original T-cell culture) with a mean of 37.2% (SD 12.0) for NKG2D-CAR and 37.3% (SD 12.2) for Empty CAR (**Figure 1D**). NKG2D-CART expression was further evaluated by staining for surface NKG2D. CD4+ T cells transduced with Empty vector (blue) did not show any NKG2D-surface expression, whereas CD8+ T cells express native NKG2D. CD4+ T cells transduced with NKG2D-CAR (red) express NKG2D at the cell surface, whereas a significant shift in MFI beyond the native NKG2D-expression is observed in CD8+ T cells bearing the NKG2D CAR (red) (**Figure 1E**). NKG2D-CART cells predominantly consisted of central memory (T_CM_) and effector memory (T_EM_) cells in both the CD4+ and CD8+ CAR subsets (**Figure 1F**) with comparable CD4:8 ratios between NKG2D-CART and Empty control T cells (**Figure 1G**). To minimize any impact of transduction efficiency on the assessment of the functional of NK2D-CAR T cells against different targets both NKG2D-CAR T cells and Empty control T cells were sorted on viable GFP+ cells prior to use in functional assays.

**Figure 2:**
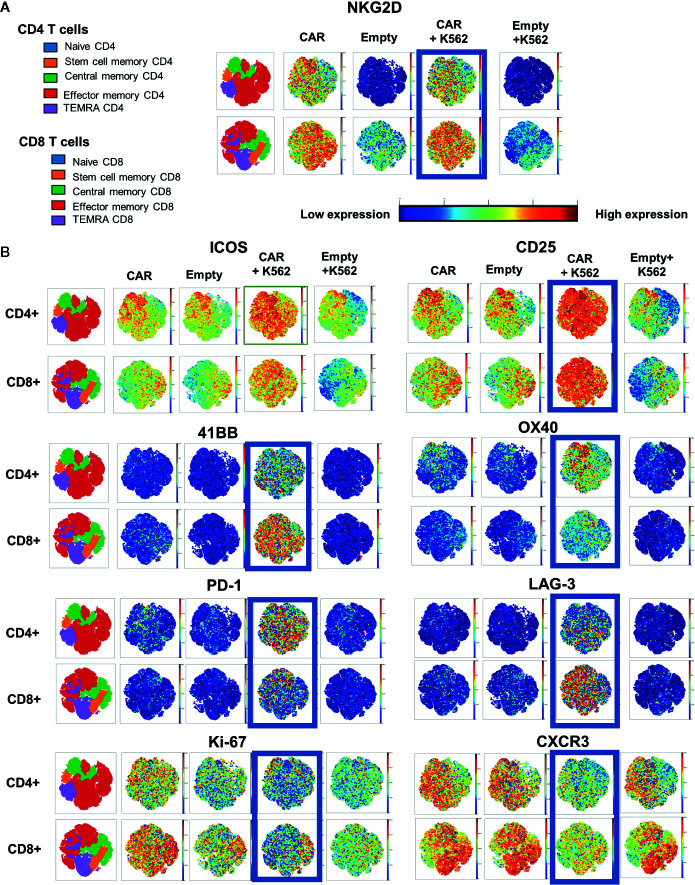
Distinct CyTOF Profile of NKG2D CAR T cells after stimulation with NKG2D-expressing targets. NKG2D CAR T cells or Empty control T cells (5 × 10^6^ cells) were co-cultured with or without K562 tumor targets (5 × 10^6^ cells) for 24 h, stained and analyzed by CyTOF Mass Cytometry to detect different characteristics of NKG2D-CAR T cells responding to NKG2D-ligand bearing leukemia cells. **(A)** Two-dimensional gating was used to identify CD45+/CD3+ CD4+ or CD8+ T cell subsets and to identify major lymphocyte subsets based on surface staining with CD45RA, CCR7 and CD95. **(A, B)** The far-left map is color-coded for each subset within the CD4+ and CD8+ populations as shown in the color legend. Surface expression of the indicated marker is displayed in representative visNE maps with a single dot representing a single analyzed cell and its location within the different phenotypic clusters. Intensity of expression of the indicated surface markers (NKG2D, ICOS, CD25, 41BB, OX40, PD-1, LAG-3, Ki-67, CXCR3) for the respective T cell conditions is represented along a color continuum as indicated, with red representing high expression and blue representing low expression.

To evaluate the characteristics of NKG2D-CAR T cells when encountering NKG2D-ligands on tumor cells, we cultured NKG2D-CAR T cells and Empty control T cells either alone or with the K562 cell line and assessed their respective expression profile by mass cytometry. This again revealed a phenotypically heterogeneous population of CAR T cells with predominance of effector memory phenotype (T_EM_) and central memory (T_CM_) populations, particularly among CD4 T cells. Distinct populations of stem cell memory (T_SCM_) and terminal effector memory (T_TEMRA_) populations were also present (**Figure 2A**). As expected, CD4 T cells transduced with Empty control vector lacked NKG2D-expression, whereas Empty control CD8 T cells exhibited expression of native NKG2D. In contrast, expression of NKG2D was high both in CD4 and CD8 CAR transduced cells (**Figure 2A**). Several surface markers were specifically upregulated on NKG2D-CAR T cells in response to NKG2D-ligand recognition on K562 targets. Whereas all T cells expressed the activation markers CD25 and ICOS following transduction and expansion, CD25 and ICOS expression was further enhanced in NKG2D-CAR T cells co-cultured with K562. In contrast, expression of CD25 and ICOS were reduced in Empty control T cells cultured with K562 (**Figure 2B**). OX-40 which is typically upregulated 18–24 h following activation, was expressed more profoundly in CD4 compared to CD8 NKG2D-CAR T cells responding to K562, whereas the costimulatory marker 41BB was more highly expressed in CD8 than CD4 NKG2D-CAR T cells. T cell activation through the NKG2D-CAR also increased expression of PD-1 and Lag-3. In this setting PD-1 was more highly expressed in CD4 NKG2D-CAR T cells while Lag3 was more highly expressed in CD8 NKG2D-CAR T cells. Expression of TIM3 was not changed after stimulation with K562 cells (data not shown). Interestingly, the proliferation marker Ki-67 and chemokine receptor CXCR3 were suppressed particularly in the T_EM_ and T_TEMRA_ NKG2D-CAR T cells in response to ligand recognition. Expression of Ki67 and CXCR3 was somewhat maintained in the T_CM_ and T_SCM_ subsets of both CD4 and CD8 NKG2D-CAR T cells.

### NKG2D-Ligands Are Frequently Expressed in AML and T-ALL

**Figure 3:**
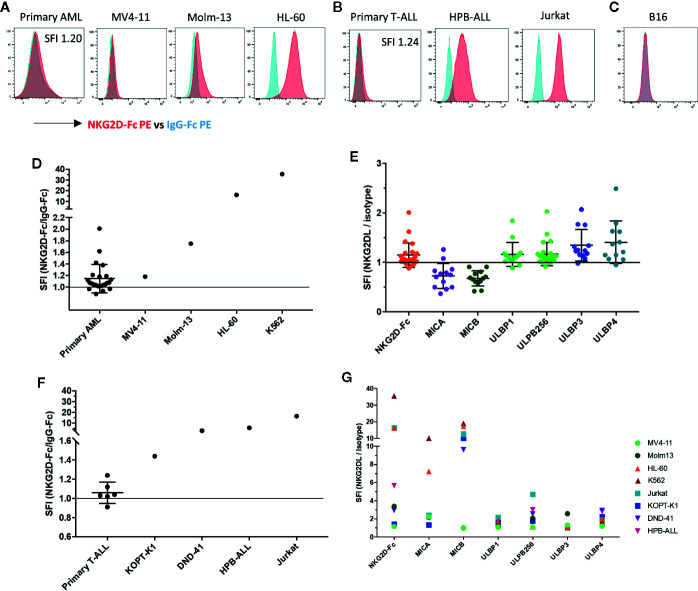
NKG2D-ligands are frequently expressed in AML and T-ALL **(A)** The NKG2D ligand surface expression in primary AML bone marrow aspirate samples and AML cell lines (MV4-11, Molm-13, HL-60) was analyzed by flow cytometry using the conjugated fusion protein NKG2D-Fc (red) and IgG-Fc isotype (blue) to evaluate ligand detection. The K562 cell line was included as a positive control due to its high level of NKG2D-ligand expression. Primary AML blasts were identified based on viability, CD45dim expression and known patient specific markers including CD117, CD34, HLA-DR, and CD33. Histograms illustrate NKG2D-Fc (red) vs. IgG-Fc (blue) isotype control staining in the respective samples **(B)** Histograms show NKG2D-Fc (red) vs. IgG-Fc (blue) isotype control staining in primary T-All blasts and T-ALL cell lines (KOPT-K1, DND-41, HPB-ALL, Jurkat). Primary T-ALL blasts were identified based on viability and a combination of CD45, CD2, CD3, CD5, CD7, CD34 and CD8 according to their patient-specific clinical expression pattern **(C)** Histogram shows NKG2D-Fc (red) vs. IgG-Fc (blue) isotype control staining in the murine melanoma cell line B16 **(D)** The intensity of NKG2D-Ligand expression is shown as specific fluorescence intensity (SFI), which represents the ratio of the mean fluorescence intensity (MFI) of NKG2D-Fc to IgG-Fc. All examined primary samples (AML n=24) and one representative sample for each of the cell lines (n ≥ 3 for each cell line) are displayed in comparison. SFI >1 is indicative of NKG2D-ligand expression above isotype **(E)** Staining of primary AML samples with available antibodies to the individual NKG2D-ligands was also undertaken and is displayed here in comparison **(F)** SFIs of all examined primary T-ALL samples (n=6) and one representative sample for each of the cell lines (n ≥ 3 for each cell line) are shown **(G)** Individual NKG2D-ligand staining with available antibodies was also performed on the cell lines indicated.

To assess the targetability of AML and T-ALL with NKG2D-ligand specific CAR T cells, we sought to evaluate the expression of NKG2D-ligands in primary AML and T-ALL in comparison to AML and T-ALL cell lines, using a conjugated NKG2D-chimeric fusion protein which mimics the ligand recognition of the NKG2D-based CAR. All evaluated AML cell lines (MV-11, Molm-13, HL-60) tested positive for NKG2D-ligand expression. The chronic myeloid leukemia cell line K562, with known high NKG2D-ligand expression was included as a positive control. The mean-fluorescence intensity (MFI) varied among different cell lines. Expression of NKG2D-ligands in primary AML blasts was lower than in most AML lines, with MV4-11 mimicking most closely the level of NKG2D-ligand expression in primary AML (**Figure 3A**). Of 24 evaluated banked bone marrow aspirate samples of patients with AML, 19 (80%) showed a specific fluorescence intensity (SFI) of >1.0, indicating expression of NKG2D-ligands above the level of the isotype control (SFI=MFI NKG2D-Fc/MFI IgG-Fc) (**Figure 3D**). The available clinical features for each sample are highlighted in **Table 2**. Primary AML samples were also stained with mAbs to individual NKG2D-ligands, with positive staining for different ULPBs (**Figure 3E**). Similarly, expression of NKG2D-Ligands was prominent in all tested T-ALL cell lines (KOPT-K1, DND-41, HPB-ALL, Jurkat), whereas NKG2D-ligands were detectable at lower levels in 5 of 6 tested primary T-ALL samples (**Figures 3B, F**), but not in the control cell line B16 (**Figure 3C**). Profiling of the individual NKG2D-ligands was also undertaken in all cell lines (**Figure 3G**).

**Table 2:** Clinical characteristics of primary AML samples.

AML Sample	Clinical Characteristics	NKG2D-L SFI
1	29yo M; 73% BM blasts; FAB M4-Eo; Complex karyotype with inversion 16. CBFB rearrangement by FISH	1.04
2	49yo F; 21% BM blasts; relapsed FLT-3 mutated AML Normal karyotype and cytogenetics	1.02
3	31yo M; 30% BM blasts; acute erythroid/myeloid leukemia 3q inversion and monosomy 7	0.97
4	45yo F, BM 76% blasts; relapsed AML s/p NMA allo-SCT; 5q deletion	1.08
5	47yo F, 91% BM blasts; secondary t-AML; FAB M1 Near tetraploidy	1.07
6	65yo M; 39% BM blasts, FAB M4 Normal cytogenetics	0.97
7	68yo F; 70% BM blasts, FAB M1 Normal cytogenetics	1.12
8	54yo F; 64% BM blasts; secondary AML arising from MDS	1.05
9	39yo F, 16% BM blasts, FAB M6 (6;9) translocation	1.02
10	55yo M, 22% BM blasts, secondary AML arising from MDS Normal cytogenetics	1.02
11	49yo F; 40% BM blasts; FAB M4 MLL rearranged, FLT-3 WT (negative)	0.88
12	60yo M, 20% BM blasts, relapsed AML, FAB M2 Normal cytogenetics	1.04
13	30yo M, 26% blasts, relapsed AML, FAB M2, FLT-3 ITD+ 13q deletion,	0.93
14	52yo M; BM 43% BM blasts, FAB M2	1.42
15	21yo M; BM 86% blasts, FAB M4 Clonal rearrangement of 7p; 9q deletion.	1.19
16	53yo M, 40% BM blasts; relapsed AML, FAB M4, FLT-3 mutated Normal cytogenetics	1.08
17	53yo M; 42% BM blasts, FAB M2 Normal cytogenetics	1.62
18	23yo F; BM blasts 70% Inversion 16	1.13
19	23yo F; BM blasts 70%; Molecular: FLT-3 ITD+, RUNX1, DNMT3 and ASXL1 mutations Normal cytogenetics	1.06
20	72yo M; 67% BM blasts Normal cytogenetics	1.38
21	67yo M; 50% BM Blasts; FAB M4; Molecular: NMP1 mutated Normal cytogenetics	1.21
22	27yo F; 82% BM blasts; molecular: CEBPA mutation Cytogenetics: trisomy 21	1.04
23	45yo M; 69% BM Blasts, FAB M4-Eo; FLT3 D835 mutation (negative for FLT3-ITD and NPM1) Inversion 16	2.01
24	64yo F; 60% BM blasts; FAB M4 Molecular: DNMT3A, WT1 mutations Cytogenetics: +8	1.18

### NKG2D CAR T Cells Exhibit Striking Activity Against AML and T-ALL Cell Lines

**Figure 4:**
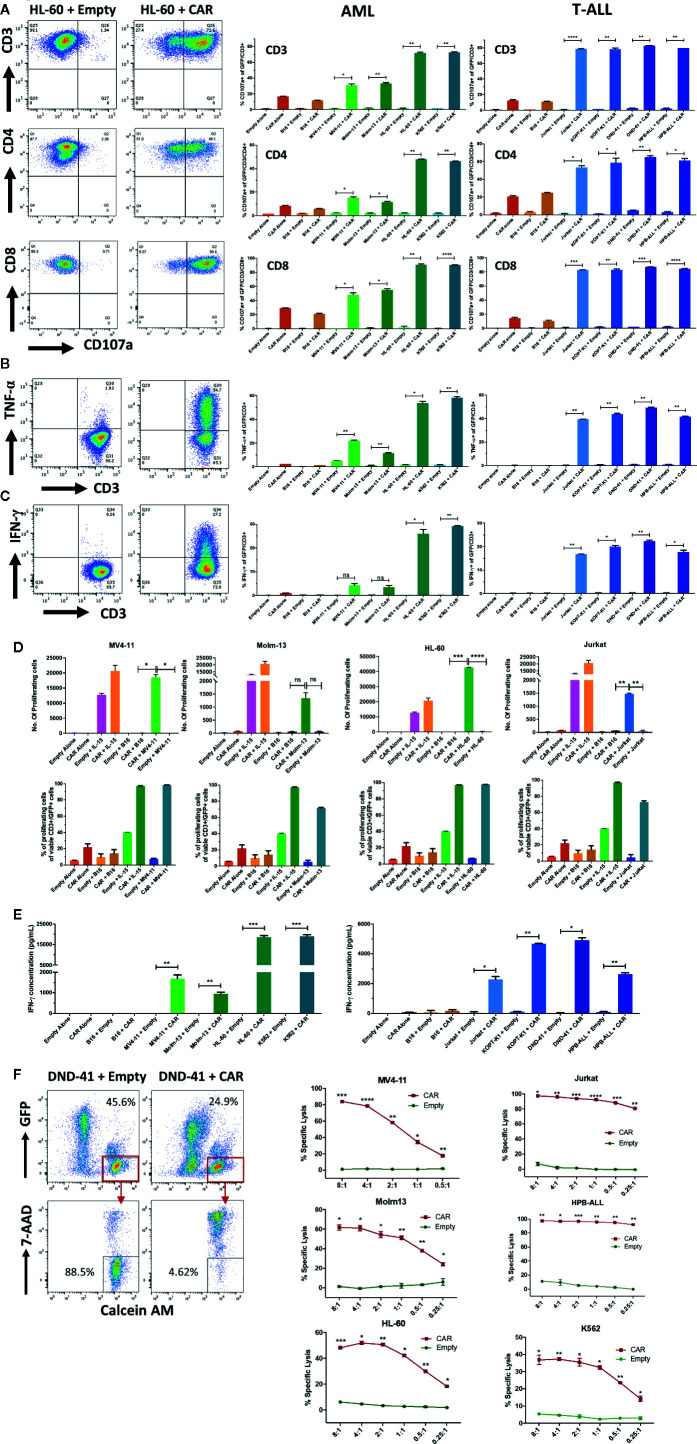
NKG2D-CAR T cells exhibit striking activity against AML and T-ALL cell lines **(A–C)** NKG2D-CAR T cells degranulate and produce TNF-α and IFN-γ in response to AML and T-ALL lines. NKG2D-CAR T cells (CAR) or primary T-cells transduced with empty vector (Empty) were cultured alone, with B16 murine melanoma cells (negative for human NKG2D-ligand expression), K562 cells (highly positive for human NKG2D-ligand expression) or the indicated AML and T-ALL cell lines at a ratio of 1:1 for 5 h. NKG2D-CAR T cells (CAR), but not control T-cells (Empty) demonstrated specific degranulation as evidenced by CD107a-positivity in response to NKG2D-ligand positive AML and T-ALL cell lines, but not to B16 negative control cell lines **(A)**. Similarly, intracellular cytokine staining revealed specific production of TNF-α **(B)** and IFN-γ **(C)** by NKG2D-CAR T cells in response to AML and T-ALL cell lines which correlated with the degree of NKG2D-ligand expression. Flow plots (left panel) illustrate an example of responses to the cell line HL-60 (gated on GFP+/CD3+ cells), whereas bar graphs (right panel) illustrate the percentage of GFP+/CD3+ cells positive for CD107a, TNF-α and IFN-γ respectively in response to the AML- and T-ALL cell lines indicated. Each experiment was performed in duplicate showing mean and Standard Deviation (SD). Welch’s t-test was utilized to detect significant differences in response. *p ≤ 0.05, **p ≤ 0.01, ***p ≤ 0.001. Results shown represent ≥ 3 independent experiments for each target **(D)** NKG2D-CAR T cells proliferate in response to NKG2D-ligand positive AML and T-ALL targets and in the presence of IL-15, but not in response to B16 negative controls, as shown by absolute # of proliferating cells calculated based on CountBright beads (top panel) and % proliferating cells (bottom panel). Each graph is representative of n ≥2 independent experiments, with each data point performed in duplicate and showing mean with SD. Welch’s t-test was utilized to detect significant differences in response. *p ≤ 0.05, **p ≤ 0.01, ***p ≤ 0.001 **(E)** NKG2D-CART cells secrete significant amounts of IFN-γ in response to AML and T-ALL cell lines. CAR and Empty T cells were co-cultured alone, with human NKG2D-ligand negative murine B16 melanoma cells, NKG2D-ligand^high^ cell line K562 or with AML and T-ALL cell lines at a ratio of 1:1 for 24 h. Cell-free supernatants were then harvested and IFN-γ secretion quantified by ELISA. Data shown is representative of n ≥ 3 independent experiments **(F)** NKG2D-CAR T cells have potent *in vitro* cytolytic activity against AML and T-ALL lines. Target cells were labeled with Calcein AM prior to co-culture with CAR or Empty T cells for 6 h at different effector:target ratios and subsequently stained with 7AAD. The representative flow plot (left panel) of DND-41 targets co-cultured with CAR or Empty at a 1:1 ratio illustrates the gating strategy to identify GFP-/Calcein AM+/7AAD- residual viable targets on the basis of which specific lysis was calculated. % Specific Lysis mediated by CAR (red lines) vs. Empty (green lines) at the indicated E:T ratios is indicated for the respective AML lines (middle panel) and T-ALL lines (right panel) as well as K562 (right panel). Each data point was performed in duplicate showing mean with SD. Each graph is representative of n ≥ independent 2 experiments except DND-41 (n=1). Each experiment was performed in duplicate or triplicate with data indicating mean with SD. Welch’s student t-test was utilized to detect significant differences. *p ≤ 0.05, **p ≤ 0.01,***p ≤ 0.001, ****p ≤ 0.0001.

We then tested the activity of the NKG2D-CAR against AML and T-ALL lines with known NKG2D-ligand expression levels. NKG2D-CAR T cells exhibited robust CD107a degranulation (**Figure 4A**) and intracellular production of TNF-α (**Figure 4B**) and IFN-γ (**Figure 4C**) when co-cultured with AML and T-ALL lines, but not in response to the murine melanoma cell line B16 (negative for human NKG2D-ligands). The response was specific to the NKG2D-CAR, as no significant degranulation or cytokine production by Empty control T cells was observed in response to tumor targets (**Figures 4A–C**). NKG2D-CAR T cells but not Empty control T cells proliferated in response to NKG2D-ligand-bearing tumor targets, but not B16 controls with IL-15 stimulated T cells serving as positive controls (**Figure 4D**). We interrogated levels of secreted IFN-γ in co-cultures of NKG2D-CAR T cells or Empty control T cells with NKG2D-ligand positive tumor cell targets, B16 or T cells alone. Similarly, to our assessment of intracellular IFN-γ production, NKG2D-CAR T cells secreted robust levels of IFN-γ in the presence of NKG2D-ligand tumor cell targets, whereas no significant IFN-γ production was detected when T cells were cultured alone or with the control cell line B16 (**Figure 4E**). Next, we evaluated the cytotoxic effects of NKG2D-CAR T cells against tumor targets compared to Empty control T cells in a flow-cytometry based cytotoxicity assay. In these studies, NKG2D-CAR T cells demonstrated robust killing of AML- and T-ALL cell line targets at E:T ratios as low as 1:1, whereas no tumor cell killing was mediated by Empty control T cells (**Figure 4F**). Cytotoxicity was not dependent on high level NKG2D-ligand expression as evidenced by effective killing of MV4-11 and Molm-13 targets (**Figures 3A, D, G** and **Figure 4F**).

### Despite Low Level NKG2D-Ligand Expression in Primary AML Samples, NKG2D-CAR T Cells Show Robust Leukemia-specific Efficacy in Functional Studies

**Figure 5:**
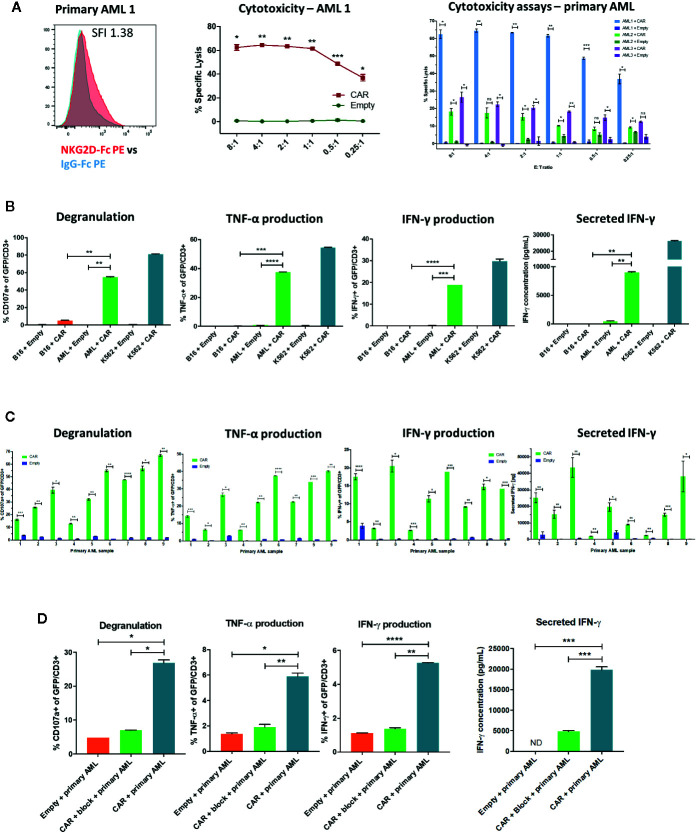
NKG2D-CAR T cells have functional activity against primary AML **(A)** NKG2D-CAR T cells but not Empty control T cells mediate a high degree of specific lysis of unsorted primary AML blasts despite low level ligand expression measured by NKG2D-Fc (SFI 1.38 for this representative sample). The degree of cytotoxicity as impacted by NKG2D-Fc SFI, blast burden, blast viability after thaw and batch of freshly manufactured CAR T cells shows some expected variability between different AML samples, but remains statistically significant compared to Empty control T cells **(B)** Despite low level NKG2D-ligand expression, NKG2D-CAR T cells but not T cells transduced with empty vector (Empty) respond to primary AML blasts (AML) with robust degranulation, intracellular production of TNF-α and IFN-γ and secretion of IFN-γ, although at lower levels than in response to the NKG2D-ligand^high^ cell line K562 **(C)** Patient-specific variability is again observed between different primary AML samples, with statistically significant differences compared to Empty control T cells **(D)** Preincubation of NKG2D-CAR T cells with NKG2D-blocking mAb abrogates the functional activity in response to primary AML, demonstrating that the effect is mediated by the CAR. Each experiment was performed in duplicate or triplicate, with data showing mean with SD. Welch’s t-test was employed to detect significant differences. *p ≤ 0.05, **p ≤ 0.01, ***p ≤ 0.001, ****p ≤ 0.0001, ns, not significant, ND, not detectable.

We next sought to evaluate the activity of NKG2D-CAR T cells against primary AML blasts in correlation with their respective NKG2D-ligand expression. Despite relatively low NKG2D-ligand expression in primary AML samples (**Figures 3D, E**, **5A**), this level of expression triggered statistically significant and robust functional NKG2D-CAR T cell responses against unsorted primary AML blasts (Pre-banking blasts percentage Mean 68.6%, SD 15.5; post-thaw viable CD45dim blast percentage Mean 91.3%, SD 6.7) including cytotoxicity (n=3) (**Figure 5A**), CD107a degranulation, intracellular IFN-γ and TNF-α production and secretion of IFN-γ (n=9) (**Figures 5B, C**). This effect was NKG2D-CAR specific as it was not mediated by Empty control T cells cultured with primary AML targets or NKG2D-CAR T cells co-cultured with the B16 control cell line (negative for human NKG2D-ligand expression). The NKG2D-ligand high-expressing cell line K562 was included as a positive control (**Figure 5B**). Moreover, these functional activities were reduced to control levels when NKG2D-CAR T cells were preincubated with NKG2D-blocking mAbs prior to coculture (**Figure 5D**).

**Figure 6:**
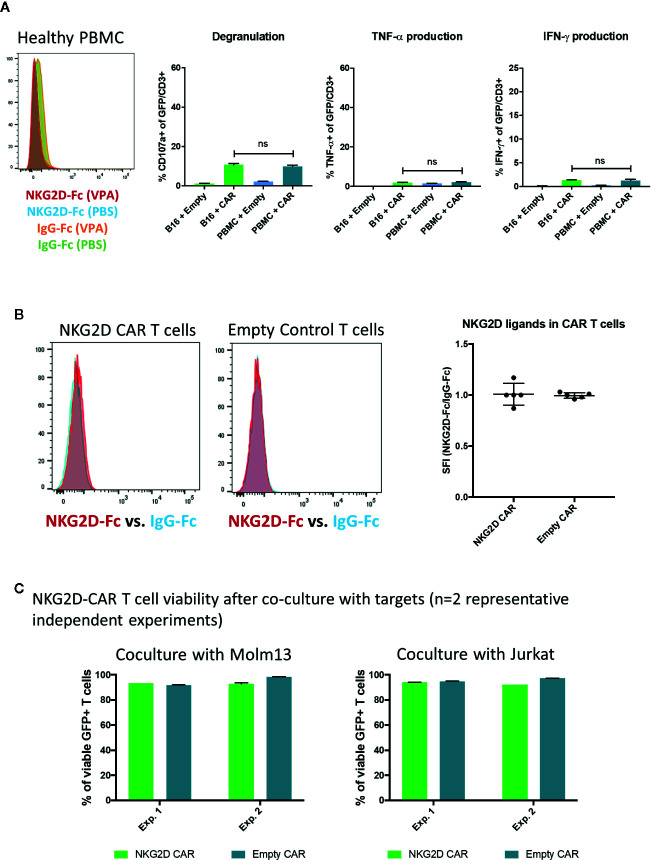
Lack of NKG2D-ligand expression on healthy PBMC or NKG2D-CAR T cells **(A)** Healthy donor PBMC were evaluated for NKG2D-ligand expression utilizing the NKG2D-Fc fusion protein and did not show any NKG2D-ligand expression (n=3). Additionally, NKG2D-CAR T cells did not trigger responses against healthy PBMC in functional assays (n=2) **(B)** Similarly, NKG2D-CAR T and Empty control cells were found to be negative for NKG2D-ligand expression (n=5) **(C)** NKG2D-CAR T cell viability was measured after 24 h co-culture with Molm13 and Jurkat targets (each n=2) and found to be ≥90%.

Importantly, healthy donor PBMC tested negative for NKG2D-Ligand expression (n=3) and did not elicit NKG2D-CAR T cell responses (n=2) (**Figure 6A**). Furthermore, no significant NKG2D-ligand expression was detected on NKG2D-CAR T cells or Empty control T cells (**Figure 6B**) at the time of use in functional assays and NKG2D-CAR T cell viability after 24 h co-culture with AML (n=2) or T-ALL (n=2) cell lines was excellent (**Figure 6C**).

### Pharmacologic HDAC Inhibition Upregulates NKG2D-Ligands in AML and Augments Antileukemic NKG2D-CAR T Cell Effects

**Figure 7:**
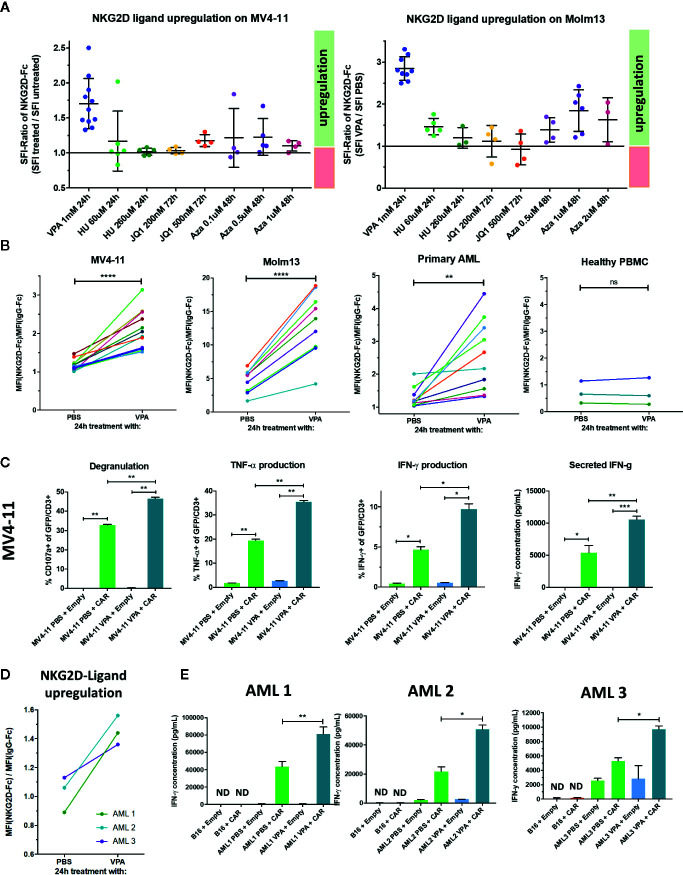
Treatment with the HDAC inhibitor Valproic acid (VPA) upregulates NKG2D-ligands in AML and enhances NKG2D-CART cell responses against AML cell lines and primary AML **(A)** The low-level NKG2D-ligand expressing cell lines MV4-11 and Molm13 were treated with Valproic acid (VPA), Hydroxyurea (HU), the bromodomain-inhibitor JQ1 or Azacitidine (Aza) at the indicated concentrations and intervals and NKG2D-ligand expression monitored by flow cytometry utilizing NKG2D-Fc and IgG-Fc staining in comparison to controls concurrently treated with PBS **(B)** Treatment with 1 mM VPA for 24 h consistently induces NKG2D-ligand upregulation in low expressing AML cell lines and primary AML, but not healthy PBMC. Graphs illustrate the difference in the SFI of cells treated with PBS versus VPA with lines connecting the SFIs of paired samples. Paired t-test was utilized to detect any significant differences in SFI between samples treated with PBS vs. VPA. *p ≤ 0.05, **p ≤ 0.01, ***p ≤ 0.001, ****p ≤ 0.0001, ns, not significant. **(C)** To determine if VPA-mediated NKG2D-ligand upregulation elicited enhanced NKG2D-CAR T cell effector function, NKG2D-CAR T cells (CAR) and control T cells (Empty) were co-cultured with the MV4-11 cell line which was either pretreated with 1 mM VPA or with PBS for 24 h prior to serving as a target. Experiments were performed in duplicate or triplicate showing mean with SD. Welch’s t-test was used to determine statistical differences *p ≤ 0.05, **p ≤ 0.01, ***p ≤ 0.001. Data shown is representative of ≥4 independent experiments **(D)** Primary AML bone marrow aspirate samples from three different patients (each containing ≥ 70% blasts) were incubated with either 1mM VPA or PBS in the presence of SCF, Flt3-ligand, GM-CSF, IL-3, and IL-6 and NKG2D-ligand expression was measured. **(E)** They were subsequently co-cultured with either NKG2D CAR T cells (CAR) or T cells transduced with empty vector (Empty) for 24 h with B16 cells serving as NKG2D-ligand negative control targets. IFN-γ secretion in the cell free supernatants was measured by ELISA. Each experiment was performed in duplicate or triplicate showing mean with SD. Welch’s t-test was employed to evaluate for statistical significance. *p ≤ 0.05, **p ≤ 0.01. Three independent experiments are shown.

Given reports of augmented NK-cell mediated antileukemic activity in AML following pharmacologic NKG2D-ligand induction, we sought to evaluate whether NKG2D-CAR T cell function may be similarly enhanced, particularly in primary AML where NKG2D-ligand expression is frequently detectable at low levels and HDAC-inhibitors and hypomethylating agents have been clinically tested (48). We therefore treated the low-level NKG2D-ligand expressing cell lines MV4-11 and Molm-13 with Valproic acid (VPA), Azacytidine (Aza), Hydroxyurea (HU) or the bromodomain-inhibitor JQ1 at different concentrations and intervals, monitored expression of NKG2D-ligands by NKG2D-Fc staining and observed at least a modest increase with all agents tested (**Figure 7A**). In these experiments, an SFI-ratio (SFI with pharmacologic agent/SFI with negative control) >1.0 indicates pharmacologic increase in NKG2D-ligand expression. After establishing best agent, dose and duration, we proceeded to treat MV4-11 (n=12), Molm13 (n=9), primary AML blasts (n=10) and healthy PBMC (n=3) with VPA or PBS at a dose of 1 mM for 24 h and evaluated NKG2D-ligand expression relative to isotype control (**Figure 7B**). We observed a statistically significant (MV4-11: p=<0.0001, Molm13: p<0.0001, primary AML: p=0.0047) increase in the SFI of cells treated with VPA compared to those treated with PBS in AML cell lines and primary AML. Importantly, no induction of NKG2D-ligand expression was observed in healthy PBMC treated with VPA (**Figure 7B**), suggesting a therapeutic window to specifically upregulate NKG2D-ligand expression in AML blasts. We then evaluated whether VPA-mediated upregulation of NKG2D-ligands enhanced anti-leukemic NKG2D- CAR T cell responses. VPA-mediated NKG2D-ligand induction significantly and consistently enhanced NKG2D-CAR T cell degranulation and cytokine production in response to both MV4-11 (**Figure 7C**) and Molm13 (data not shown) (each, n≥4) pretreated with VPA. Most importantly, this effect was also observed in primary AML samples, where VPA-mediated NKG2D-ligand upregulation (**Figure 7D**) was a successful strategy to significantly enhance NKG2D-CAR T cell-mediated IFN-γ production (n=3) (**Figure 7E**).

## Discussion

Safe and effective CAR T cell strategies for the treatment of AML and T-ALL represent an unmet medical need. NKG2D-ligands are not expressed on healthy T cells or freshly isolated CD34+ stem cells (53) and therefore represent an alternative CAR T cell target for the treatment of these high risk leukemias. One potential toxicity concern when targeting NKG2D-ligands is the possibility of ligand-upregulation on healthy cells and the induction of an inflammatory feedback loop. No myelotoxicity, T-cell toxicity or other autoimmune effects were observed in a recent Phase 1 trial of NKG2D-CAR T cells in adults with AML and multiple myeloma, providing a first reassuring safety signal of this approach in the clinic (40). However, no objective responses were observed. Since NKG2D-CAR efficacy had not been examined in preclinical models of AML, this led us to interrogate whether NKG2D-ligands are targets worthy of further pursuit in AML. NKG2D-ligands were detected on AML blasts in the trial, but at lower levels than targets with established clinical efficacy such as CD19 **(** 54) or CD22 (55). This raised the question whether NKG2D-ligand expression observed on the trial was sufficient to expect clinical efficacy and whether NKG2D-ligand expression in patients, who had sufficiently stable disease to await CAR T cell manufacture without intercurrent chemotherapy was generally representative for AML or not. To address these questions and establish NKG2D-ligand expression patterns in T-ALL, we investigated NKG2D-ligand expression in a larger cohort of primary AML and T-ALL samples. Consistent with other reports in the literature **(** 22, 43, 56), we confirm here that NKG2D-ligands are frequently expressed in primary AML and T-ALL and demonstrate robust NKG2D-ligand expression in different AML and T-ALL cell lines. However, primary AML and T-ALL express NKG2D-ligands at lower levels than most AML and T-ALL cell lines.

To investigate the efficacy of NKG2D-CARs in *in vitro* models, we generated primary human NKG2D-CAR T cells and compared them to control T-cells in functional readouts assessing CAR T cell degranulation, cytokine production, proliferation and cytotoxicity. We observed potent NKG2D-CAR T cell responses to AML and T-ALL cell lines and showed that these were mediated specifically by the NKG2D-CAR interaction with NKG2D-ligands, given that similar effects were not mediated by control T-cells, were abrogated by preincubation of NGK2D-CAR T cells with NKG2D-blocking mAb and were not observed in response to NKG2D-ligand negative targets. We also observed that higher levels of NKG2D-ligand expression correlated with higher levels of degranulation and cytokine production although it did not appear to impact NKG2D-CAR T cell mediated killing of targets which was consistently efficient at very low E:T ratios. Notably, healthy donor PBMC did not express NKG2D-ligands or elicit NKG2D-CAR T cell responses. We examined NKG2D-ligand expression on NKG2D-CAR T cell and control T cells at the time they were utilized in functional assays (end of manufacture) and observed no NKG2D-ligand expression on NKG2D-CAR T cells at this time (57). We examined the phenotype of NKG2D-CAR T cells, which consisted predominantly of T_CM_ and T_EM_ T cells mediating antileukemic effector functions, but also contained T_SCM_ with capacity for CAR T cell regeneration and longevity. Mass-cytometric analysis revealed a distinct signature of NKG2D-CAR T cells responding to NKG2D-ligand positive leukemia targets when compared to Empty control T cells or NKG2D-CAR T cells lacking NKG2D-ligand recognition. NKG2D-CAR T cells showed significant upregulation of the activation markers ICOS and CD25 in response to K562. Similarly, the costimulatory molecules 41BB and OX-40 were upregulated in NKG2D-CAR T cells with natural predilection in CD8 and CD4 T cells respectively. PD-1 and Lag-3 were similarly upregulated selectively in tumor-recognizing NKG2D-CAR T cells, which may be more reflective of T-cell activation than exhaustion, given robust NKG2D-CAR functionality observed in several assays, but suggests that NKG2D-CAR T cell response could be further enhanced in combination with checkpoint blockade or genetic modification strategies. Interestingly, expression of the proliferation marker Ki67 and homing marker CXCR3 was decreased in the T_EM_ and T_EMRA_ CAR subsets involved in the active anti-tumor response while they were relatively maintained in the T_SCM_ and T_CM_ subsets responsible for maintaining an ongoing NKG2D-CAR T cell response.

Based on prior reports of enhanced NK cell-mediated AML responses associated with NKG2D-ligand induction by HDAC inhibitors (46, 58), we hypothesized that selective enhancement of NKG2D-ligand expression in AML could be achieved pharmacologically and lead to enhanced NKG2D-CAR efficacy in this indication. After screening different agents and conditions, we established that NKG2D-ligand expression could be consistently increased in low-level expressing AML cell lines and in primary AML blasts after pretreatment with the HDAC inhibitor valproate and that this strategy significantly enhanced NKG2D-CAR anti-tumor responses against AML. Importantly, NKG2D-ligand expression was enhanced in AML blasts with low ligand expression, but not NKG2D-ligand negative healthy PBMC. Given that epigenetic therapeutics, including HDAC-inhibitors are in clinical use in AML, our data supports the further investigation of NKG2D-CAR T cell therapy in conjunction with pharmacologic HDAC-inhibition in xenograft models of AML as a rational combination therapy with synergistic anti-leukemia effects. Such therapy may not only augment direct anti-tumor effects mediated by NKG2D-CAR T cells, but also enhance the ability of NKG2D-CAR T cells to expand against antigen. In summary, we provide comprehensive preclinical evidence of *in vitro* NKG2D-CAR T cell efficacy in T-ALL and AML and demonstrate that pharmacologic HDAC-inhibition achieves selective upregulation of NKG2D-ligands on the surface of AML blasts which in turn leads to enhanced NKG2D-CAR T cell efficacy.

## Data Availability Statement

The original contributions presented in the study are included in the article/supplementary materials. Further inquiries can be directed to the corresponding author.

## Ethics Statement

The studies involving human participants were reviewed and approved by Dana-Farber Cancer Institute Institutional Review Board. The patients/participants provided their written informed consent to participate in this study.

## Author Contributions

SB, GD, and JR designed the experiments. SB and LD conducted the experiments, analyzed the data, and wrote the manuscript. This work is part of LD’s doctoral thesis. KR conducted bioinformatic analyses. JG and YK conducted experiments and analyses. All authors contributed to the article and approved the submitted version.

## Funding

Leukemia and Lymphoma Society Specialized Center of Research LLS SCOR 7009-12 (JR and GD), German Academic Scholarship Foundation (LD), and Alex’s Lemonade Stand Foundation Centers of Excellence grant (SB).

## Conflict of Interest

GD is currently an employee of Novartis and owns Novartis stock. JR reports research funding from Amgen, Equillium, and Kite Pharma, and consulting income from Avrobio, Falcon Therapeutics, Infinity Pharmaceuticals, LifeVault Bio, Rheos Medicines, Talaris Therapeutics and TScan Therapeutics.

The remaining authors declare that the research was conducted in the absence of any commercial or financial relationships that could be construed as a potential conflict of interest.

## References

[1] Thein MS Ershler WB Jemal A Yates JW Baer MR Outcome of older patients with acute myeloid leukemia: an analysis of SEER data over 3 decades. Cancer (2013) 119(15):2720–7. 10.1002/cncr.28129 PMC3821042 23633441

[2] Appelbaum FR Gundacker H Head DR Slovak ML Willman CL Godwin JE Age and acute myeloid leukemia. Blood (2006) 107(9):3481–5. 10.1182/blood-2005-09-3724 PMC1895766 16455952

[3] Zwaan CM Kolb EA Reinhardt D Abrahamsson J Adachi S Aplenc R Collaborative Efforts Driving Progress in Pediatric Acute Myeloid Leukemia. J Clin Oncol (2015) 33(27):2949–62. 10.1200/JCO.2015.62.8289 PMC4567700 26304895

[4] Guru Murthy GS Pondaiah SK Abedin S Atallah E Incidence and survival of T-cell acute lymphoblastic leukemia in the United States. Leuk Lymphoma (2019) 60(5):1171-8. 10.1080/10428194.2018.1522442 30407885

[5] Kochenderfer JN Somerville RPT Lu T Yang JC Sherry RM Feldman SA Long-Duration Complete Remissions of Diffuse Large B Cell Lymphoma after Anti-CD19 Chimeric Antigen Receptor T Cell Therapy. Mol Ther J Am Soc Gene Ther (2017) 25(10):2245–53. 10.1016/j.ymthe.2017.07.004 PMC5628864 28803861

[6] Maude SL Laetsch TW Buechner J Rives S Boyer M Bittencourt H Tisagenlecleucel in Children and Young Adults with B-Cell Lymphoblastic Leukemia. N Engl J Med (2018) 378(5):439–48. 10.1056/NEJMoa1709866 PMC5996391 29385370

[7] Park JH Riviere I Gonen M Wang X Senechal B Curran KJ Long-Term Follow-up of CD19 CAR Therapy in Acute Lymphoblastic Leukemia. N Engl J Med (2018) 378(5):449–59. 10.1056/NEJMoa1709919 PMC6637939 29385376

[8] Gardner RA Finney O Annesley C Brakke H Summers C Leger K Intent-to-treat leukemia remission by CD19 CAR T cells of defined formulation and dose in children and young adults. Blood (2017) 129(25):3322–31. 10.1182/blood-2017-02-769208 PMC5482103 28408462

[9] Neelapu SS Locke FL Bartlett NL Lekakis LJ Miklos DB Jacobson CA Axicabtagene Ciloleucel CAR T-Cell Therapy in Refractory Large B-Cell Lymphoma. N Engl J Med (2017) 377(26):2531–44. 10.1056/NEJMoa1707447 PMC5882485 29226797

[10] Turtle CJ Hanafi LA Berger C Hudecek M Pender B Robinson E Immunotherapy of non-Hodgkin’s lymphoma with a defined ratio of CD8+ and CD4+ CD19-specific chimeric antigen receptor-modified T cells. Sci Trans Med (2016) 8(355):355ra116. 10.1126/scitranslmed.aaf8621 PMC5045301 27605551

[11] Cummins KD Gill S Anti-CD123 chimeric antigen receptor T-cells (CART): an evolving treatment strategy for hematological malignancies, and a potential ace-in-the-hole against antigen-negative relapse. Leuk Lymphoma (2018) 59(7):1539–53. 10.1080/10428194.2017.1375107 28901790

[12] Lynn RC Poussin M Kalota A Feng Y Low PS Dimitrov DS Targeting of folate receptor beta on acute myeloid leukemia blasts with chimeric antigen receptor-expressing T cells. Blood (2015) 125(22):3466–76. 10.1182/blood-2014-11-612721 PMC4447861 25887778

[13] Tasian SK Acute myeloid leukemia chimeric antigen receptor T-cell immunotherapy: how far up the road have we traveled? Ther Adv Hematol (2018) 9(6):135–48. 10.1177/2040620718774268 PMC5992809 29899889

[14] Kenderian SS Ruella M Shestova O Klichinsky M Aikawa V Morrissette JJ CD33-specific chimeric antigen receptor T cells exhibit potent preclinical activity against human acute myeloid leukemia. Leukemia (2015) 29(8):1637–47. 10.1038/leu.2015.52 PMC4644600 25721896

[15] Kim MY Yu KR Kenderian SS Ruella M Chen S Shin TH Genetic Inactivation of CD33 in Hematopoietic Stem Cells to Enable CAR T Cell Immunotherapy for Acute Myeloid Leukemia. Cell (2018) 173(6):1439–53.e19. 10.1016/j.cell.2018.05.013 29856956 PMC6003425

[16] McKoy JM Angelotta C Bennett CL Tallman MS Wadleigh M Evens AM Gemtuzumab ozogamicin-associated sinusoidal obstructive syndrome (SOS): an overview from the research on adverse drug events and reports (RADAR) project. Leuk Res (2007) 31(5):599–604. 10.1016/j.leukres.2006.07.005 16959316

[17] Gomes-Silva D Srinivasan M Sharma S Lee CM Wagner DL Davis TH CD7-edited T cells expressing a CD7-specific CAR for the therapy of T-cell malignancies. Blood (2017) 130(3):285–96. 10.1182/blood-2017-01-761320 PMC5520470 28539325

[18] Png YT Vinanica N Kamiya T Shimasaki N Coustan-Smith E Campana D Blockade of CD7 expression in T cells for effective chimeric antigen receptor targeting of T-cell malignancies. Blood Adv (2017) 1(25):2348–60. 10.1182/bloodadvances.2017009928 PMC5729624 29296885

[19] Lanier LL NKG2D Receptor and Its Ligands in Host Defense. Cancer Immunol Res (2015) 3(6):575–82. 10.1158/2326-6066.CIR-15-0098 PMC4457299 26041808

[20] Spear P Wu MR Sentman ML Sentman CL NKG2D ligands as therapeutic targets. Cancer Immunity (2013) 13:8. 23833565 PMC3700746

[21] Groh V Rhinehart R Secrist H Bauer S Grabstein KH Spies T Broad tumor-associated expression and recognition by tumor-derived gamma delta T cells of MICA and MICB. Proc Natl Acad Sci USA (1999) 96(12):6879–84. 10.1073/pnas.96.12.6879 PMC22010 10359807

[22] Hilpert J Grosse-Hovest L Grunebach F Buechele C Nuebling T Raum T Comprehensive analysis of NKG2D ligand expression and release in leukemia: implications for NKG2D-mediated NK cell responses. J Immunol (2012) 189(3):1360–71. 10.4049/jimmunol.1200796 22730533

[23] Barber A Meehan KR Sentman CL Treatment of multiple myeloma with adoptively transferred chimeric NKG2D receptor-expressing T cells. Gene Ther (2011) 18(5):509–16. 10.1038/gt.2010.174 PMC3095961 21209626

[24] Barber A Zhang T Megli CJ Wu J Meehan KR Sentman CL Chimeric NKG2D receptor-expressing T cells as an immunotherapy for multiple myeloma. Exp Hematol (2008) 36(10):1318–28. 10.1016/j.exphem.2008.04.010 PMC2638591 18599182

[25] Barber A Zhang T Sentman CL Immunotherapy with chimeric NKG2D receptors leads to long-term tumor-free survival and development of host antitumor immunity in murine ovarian cancer. J Immunol (2008) 180(1):72–8. 10.4049/jimmunol.180.1.72 18097006

[26] Zhang T Barber A Sentman CL Chimeric NKG2D modified T cells inhibit systemic T-cell lymphoma growth in a manner involving multiple cytokines and cytotoxic pathways. Cancer Res (2007) 67(22):11029–36. 10.1158/0008-5472.CAN-07-2251 18006849

[27] Barber A Zhang T DeMars LR Conejo-Garcia J Roby KF Sentman CL Chimeric NKG2D receptor-bearing T cells as immunotherapy for ovarian cancer. Cancer Res (2007) 67(10):5003–8. 10.1158/0008-5472.CAN-06-4047 17510432

[28] Zhang T Barber A Sentman CL Generation of antitumor responses by genetic modification of primary human T cells with a chimeric NKG2D receptor. Cancer Res (2006) 66(11):5927–33. 10.1158/0008-5472.CAN-06-0130 16740733

[29] Zhang T Lemoi BA Sentman CL Chimeric NK-receptor-bearing T cells mediate antitumor immunotherapy. Blood (2005) 106(5):1544–51. 10.1182/blood-2004-11-4365 PMC1895219 15890688

[30] Fernandez L Metais JY Escudero A Vela M Valentin J Vallcorba I Memory T Cells Expressing an NKG2D-CAR Efficiently Target Osteosarcoma Cells. Clin Cancer Res (2017) 23(19):5824–35. 10.1158/1078-0432.CCR-17-0075 28659311

[31] Han Y Xie W Song DG Powell DJ Jr. Control of triple-negative breast cancer using ex vivo self-enriched, costimulated NKG2D CAR T cells. J Hematol Oncol (2018) 11(1):92. 10.1186/s13045-018-0635-z 29980239 PMC6035420

[32] Tao K He M Tao F Xu G Ye M Zheng Y Development of NKG2D-based chimeric antigen receptor-T cells for gastric cancer treatment. Cancer Chemother Pharmacol (2018) 82(5):815–27. 10.1007/s00280-018-3670-0 30132099

[33] Spear P Barber A Rynda-Apple A Sentman CL NKG2D CAR T-cell therapy inhibits the growth of NKG2D ligand heterogeneous tumors. Immunol Cell Biol (2013) 91(6):435–40. 10.1038/icb.2013.17 PMC3700668 23628805

[34] Parihar R Rivas C Huynh M Omer B Lapteva N Metelitsa LS NK Cells Expressing a Chimeric Activating Receptor Eliminate MDSCs and Rescue Impaired CAR-T Cell Activity against Solid Tumors. Cancer Immunol Res (2019) 7(3):363–75. 10.1158/2326-6066.CIR-18-0572 PMC7906796 30651290

[35] Hansen CH Holm TL Krych L Andresen L Nielsen DS Rune I Gut microbiota regulates NKG2D ligand expression on intestinal epithelial cells. Eur J Immunol (2013) 43(2):447–57. 10.1002/eji.201242462 23136011

[36] Hue S Mention JJ Monteiro RC Zhang S Cellier C Schmitz J A direct role for NKG2D/MICA interaction in villous atrophy during celiac disease. Immunity (2004) 21(3):367–77. 10.1016/j.immuni.2004.06.018 15357948

[37] VanSeggelen H Hammill JA Dvorkin-Gheva A Tantalo DG Kwiecien JM Denisova GF T Cells Engineered With Chimeric Antigen Receptors Targeting NKG2D Ligands Display Lethal Toxicity in Mice. Mol Ther J Am Soc Gene Ther (2015) 23(10):1600–10. 10.1038/mt.2015.119 PMC4817919 26122933

[38] Sentman ML Murad JM Cook WJ Wu MR Reder J Baumeister SH Mechanisms of Acute Toxicity in NKG2D Chimeric Antigen Receptor T Cell-Treated Mice. J Immunol (2016) 197(12):4674–85. 10.4049/jimmunol.1600769 PMC5136298 27849169

[39] Murad JM Baumeister SH Werner L Daley H Trebeden-Negre H Reder J Manufacturing development and clinical production of NKG2D chimeric antigen receptor-expressing T cells for autologous adoptive cell therapy. Cytotherapy (2018) 20(7):952–63. 10.1016/j.jcyt.2018.05.001 PMC6127861 30180944

[40] Baumeister SH Murad J Werner L Daley H Trebeden-Negre H Gicobi JK Phase 1 Trial of Autologous CAR T Cells Targeting NKG2D Ligands in Patients with AML/MDS and Multiple Myeloma. Cancer Immunol Res (2019) 7(1):100–12. 10.1158/2326-6066.CIR-18-0307 PMC7814996 30396908

[41] Schlegel P Ditthard K Lang P Mezger M Michaelis S Handgretinger R NKG2D Signaling Leads to NK Cell Mediated Lysis of Childhood AML. J Immunol Res (2015) 2015:473175. 10.1155/2015/473175 26236752 PMC4510257

[42] Salih HR Antropius H Gieseke F Lutz SZ Kanz L Rammensee HG Functional expression and release of ligands for the activating immunoreceptor NKG2D in leukemia. Blood (2003) 102(4):1389–96. 10.1182/blood-2003-01-0019 12714493

[43] Sanchez-Correa B Morgado S Gayoso I Bergua JM Casado JG Arcos MJ Human NK cells in acute myeloid leukaemia patients: analysis of NK cell-activating receptors and their ligands. Cancer Immunol Immunother CII (2011) 60(8):1195–205. 10.1007/s00262-011-1050-2 PMC11028638 21644031

[44] Mastaglio S Wong E Perera T Ripley J Blombery P Smyth MJ Natural killer receptor ligand expression on acute myeloid leukemia impacts survival and relapse after chemotherapy. Blood Adv (2018) 2(4):335–46. 10.1182/bloodadvances.2017015230 PMC5858482 29449224

[45] Pende D Spaggiari GM Marcenaro S Martini S Rivera P Capobianco A Analysis of the receptor-ligand interactions in the natural killer-mediated lysis of freshly isolated myeloid or lymphoblastic leukemias: evidence for the involvement of the Poliovirus receptor (CD155) and Nectin-2 (CD112). Blood (2005) 105(5):2066–73. 10.1182/blood-2004-09-3548 15536144

[46] Diermayr S Himmelreich H Durovic B Mathys-Schneeberger A Siegler U Langenkamp U NKG2D ligand expression in AML increases in response to HDAC inhibitor valproic acid and contributes to allorecognition by NK-cell lines with single KIR-HLA class I specificities. Blood (2008) 111(3):1428–36. 10.1182/blood-2007-07-101311 17993609

[47] Rohner A Langenkamp U Siegler U Kalberer CP Wodnar-Filipowicz A Differentiation-promoting drugs up-regulate NKG2D ligand expression and enhance the susceptibility of acute myeloid leukemia cells to natural killer cell-mediated lysis. Leuk Res (2007) 31(10):1393–402. 10.1016/j.leukres.2007.02.020 17391757

[48] Bewersdorf JP Shallis R Stahl M Zeidan AM Epigenetic therapy combinations in acute myeloid leukemia: what are the options? Ther Adv Hematol (2019) 10:2040620718816698. 10.1177/2040620718816698 30719265 PMC6348528

[49] Soriano AO Yang H Faderl S Estrov Z Giles F Ravandi F Safety and clinical activity of the combination of 5-azacytidine, valproic acid, and all-trans retinoic acid in acute myeloid leukemia and myelodysplastic syndrome. Blood (2007) 110(7):2302–8. 10.1182/blood-2007-03-078576 17596541

[50] Walter RB Medeiros BC Gardner KM Orlowski KF Gallegos L Scott BL Gemtuzumab ozogamicin in combination with vorinostat and azacitidine in older patients with relapsed or refractory acute myeloid leukemia: a phase I/II study. Haematologica (2014) 99(1):54–9. 10.3324/haematol.2013.096545 PMC4007917 24142996

[51] Newrzela S Gunda B von Laer D T cell culture for gammaretroviral transfer. Methods Mol Biol (2009) 506:71–82. 10.1007/978-1-59745-409-4_6 19110620

[52] Hirakawa M Matos TR Liu H Koreth J Kim HT Paul NE Low-dose IL-2 selectively activates subsets of CD4(+) Tregs and NK cells. JCI Insight (2016) 1(18):e89278. 10.1172/jci.insight.89278 27812545 PMC5085610

[53] Guilloton F de Thonel A Jean C Demur C Mansat-De Mas V Laurent G TNFalpha stimulates NKG2D-mediated lytic activity of acute myeloid leukemic cells. Leukemia (2005) 19(12):2206–14. 10.1038/sj.leu.2403952 16239914

[54] Grupp SA Kalos M Barrett D Aplenc R Porter DL Rheingold SR Chimeric antigen receptor-modified T cells for acute lymphoid leukemia. N Engl J Med (2013) 368(16):1509–18. 10.1056/NEJMoa1215134 PMC4058440 23527958

[55] Fry TJ Shah NN Orentas RJ Stetler-Stevenson M Yuan CM Ramakrishna S CD22-targeted CAR T cells induce remission in B-ALL that is naive or resistant to CD19-targeted CAR immunotherapy. Nat Med (2018) 24(1):20–8. 10.1038/nm.4441 PMC5774642 29155426

[56] Nowbakht P Ionescu MC Rohner A Kalberer CP Rossy E Mori L Ligands for natural killer cell-activating receptors are expressed upon the maturation of normal myelomonocytic cells but at low levels in acute myeloid leukemias. Blood (2005) 105(9):3615–22. 10.1182/blood-2004-07-2585 15657183

[57] Breman E Demoulin B Agaugue S Mauen S Michaux A Springuel L Overcoming Target Driven Fratricide for T Cell Therapy. Front Immunol (2018) 9:2940. 10.3389/fimmu.2018.02940 30619300 PMC6299907

[58] Poggi A Catellani S Garuti A Pierri I Gobbi M Zocchi MR Effective in vivo induction of NKG2D ligands in acute myeloid leukaemias by all-trans-retinoic acid or sodium valproate. Leukemia (2009) 23(4):641–8. 10.1038/leu.2008.354 19151770

